# Decoding pathogenesis factors involved in the progression of ATLL or HAM/TSP after infection by HTLV-1 through a systems virology study

**DOI:** 10.1186/s12985-021-01643-8

**Published:** 2021-08-26

**Authors:** Mohadeseh Zarei Ghobadi, Rahman Emamzadeh, Majid Teymoori-Rad, Sayed-Hamidreza Mozhgani

**Affiliations:** 1 Department of Cell and Molecular Biology and Microbiology, Faculty of Biological Science and Technology, University of Isfahan, Isfahan, Iran; 2 Department of Virology, School of Public Health, Tehran University of Medical Sciences, Tehran, Iran; 3 Department of Microbiology, School of Medicine, Alborz University of Medical Sciences, Karaj, Iran; 4 Non‑Communicable Diseases Research Center, Alborz University of Medical Sciences, Karaj, Iran

**Keywords:** HTLV-1, ACs, ATLL, HAM/TSP, Differentially co-expressed modules, Pathogenesis

## Abstract

**Background:**

Human T-cell Leukemia Virus type-1 (HTLV-1) is a retrovirus that causes two diseases including Adult T-cell Leukemia/Lymphoma (ATLL cancer) and HTLV-1 Associated Myelopathy/Tropical Spastic Paraparesis (HAM/TSP, a neurodegenerative disease) after a long latency period as an asymptomatic carrier (AC). There are no obvious explanations about how each of the mentioned diseases develops in the AC carriers. Finding the discriminative molecular factors and pathways may clarify the destiny of the infection.

**Methods:**

To shed light on the involved molecular players and activated pathways in each state, differentially co-expressed modules (DiffCoEx) algorithm was employed to identify the highly correlated genes which were co-expressed differently between normal and ACs, ACs and ATLL, as well as ACs and HAM/TSP samples. Through differential pathway analysis, the dysregulated pathways and the specific disease-genes-pathways were figured out. Moreover, the common genes between the member of DiffCoEx and differentially expressed genes were found and the specific genes in ATLL and HAM/TSP were introduced as possible biomarkers.

**Results:**

The dysregulated genes in the ATLL were mostly enriched in immune and cancer-related pathways while the ones in the HAM/TSP were enriched in immune, inflammation, and neurological pathways. The differential pathway analysis clarified the differences between the gene players in the common activated pathways. Eventually, the final analysis revealed the involvement of specific dysregulated genes including *KIRREL2*, *RAB36*, and *KANK1* in HAM/TSP as well as *LTB4R2*, *HCN4*, *FZD9*, *GRIK5*, *CREB3L4*, *TACR2*, *FRMD1*, LHB*, FGF3*, *TEAD3*, *GRIN2D*, *GNRH2*, *PRLH*, *GPR156*, and *CRHR2* in ATLL.

**Conclusion:**

The identified potential prognostic biomarkers and therapeutic targets are proposed as the most important platers in developing ATLL or HAM/TSP. Moreover, the proposed signaling network clarifies the differences between the functional players in the activated pathways in ACs, ATLL, and HAM/TSP.

**Supplementary Information:**

The online version contains supplementary material available at 10.1186/s12985-021-01643-8.

## Introduction

Human T-cell Leukemia Virus type-1 (HTLV-1) belongs to the family Retroviridae and subfamily Orthoretrovirinae [1]. The HTLV-1 infection may cause developing two major diseases including Adult T-cell Leukemia/Lymphoma (ATLL cancer) and Tropical Spastic Paraparesis/HTLV-1 Associated Myelopathy (TSP/HAM) after elapsing an asymptomatic carrier (AC) state. Although HTLV-1 is not a widespread virus all over the world, it is an endemic pathogen in sub-Saharan Africa, East north of Iran, the Caribbean region, Japan, and South America [2]. Approximately, 90% of the infected HTLV-1 are asymptomatic carriers (ACs) [3] with the capability of the silent transmission of the virus through blood contact, sexual intercourse, breastfeeding, etc.[4].

There are three main receptors on the cells for HTLV-1 including neuropilin, glucose transporter 1 (GLUT-1), and heperan sulfate proteoglycan [5]. The HTLV-1 proviral DNA is found in the immune cell types comprising dendritic cells, monocytes, CD8+ T-cells, B cells, and in higher extent in CD4+ T-cells [6, 7]. Two oncogenic proteins named as the HTLV-1 basic leucine zipper protein (HBZ) and the transactivator protein (Tax) are frequently expressed by the HTLV-1 genome. However, the tax gene expresses in only around 40% of ATLL patients [8]. Tax has a critical function in the viral pathogenesis of HAM/TSP patients through promoting the proliferation of infected cells by activating NFκB and AP-1 pathways, avoiding apoptosis, and activating cytotoxic T lymphocyte (CTL) response [3, 9]. On the other hand, HBZ prohibits NF-κB pathway, promotes tumor progression, and boosts T cell proliferation and lymphoma [10, 11].

One of the beneficial approaches to find the patterns of co-regulated genes is gene co-expression analysis [12]. Two common approaches are usually utilized to determine the mechanistic diversity between two conditions including identifying differential gene expression and differential gene co-expression. Through co-expression analysis, the highly correlated genes (a module) can be identified. Moreover, the differential co-expressed analysis determines the specific co-regulated genes in each condition. DiffCoEx is a sensitive and efficient method to find the gene co-expression differences which are grouped in various modules between multiple conditions [13]. The major advantage of this method is determining significant differential co-expressed gene groups even in the attendance of the within-group correlation across two conditions.

Since the pathogenesis mechanism of HTLV-1 as a virus-caused cancer and virus-caused neurologic disease have not been yet fully ascertained, the identification of differential co-regulated genes can determine new functional players in ACs, ATLL, and HAM/TSP. To this end, we integrated various gene expression datasets and then found the differential co-expressed genes between every two conditions employing DiffCoEx approach. The outcomes specified the gene players and activated pathways implicated in the development of each disease after infection.

## Methods

### Data collection, merging, and preprocessing

**Fig. 1:**
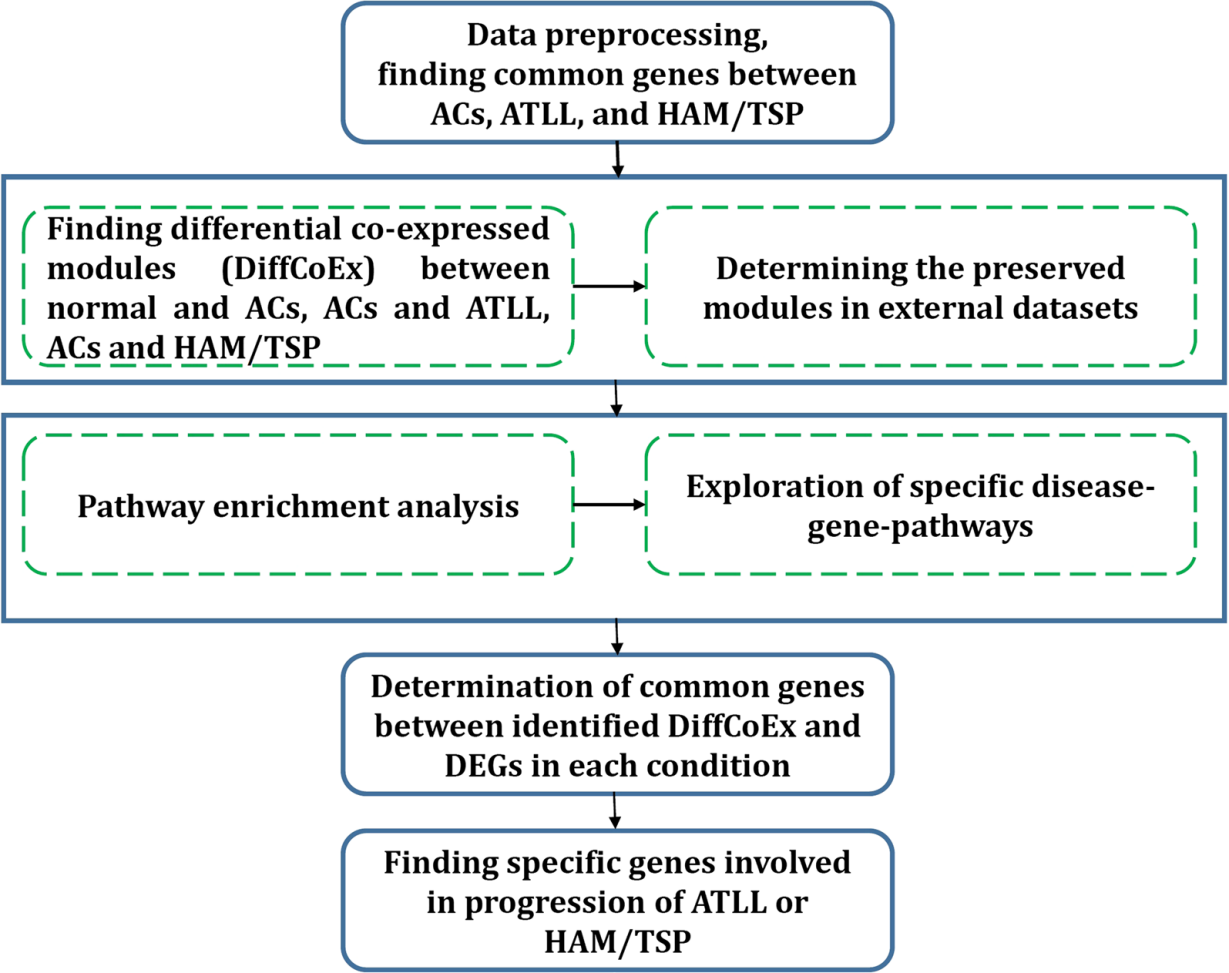
Workflow of the proposed method

To find the relevant datasets correspond to our study purpose, Gene Expression Omnibus (GEO) repository database was searched. A total of four microarray datasets including GSE29312, GSE29332, GSE33615, and GSE55851 were selected to be involved in our analysis. In total, these datasets contain 58 normal samples, 43 ACs samples, 62 ATLL samples, and 20 HAM/TSP samples. Since datasets belong to different platforms, removeBatchEffect function in the R limma package was utilized to remove the batch effect across different datasets [14]. The expression data of samples related to each condition were merged, individually. A total of 15,565 common genes were finally used for further analysis. The merged data were quantile-normalized and log2-transformed. Moreover, to validate the identified modules, the ACs and HAM/TSP samples from GSE38537 dataset and ATLL samples from GSE43017 dataset were used. Table 1 contains more details related to each dataset. Moreover, Fig. 1 indicates the workflow of the utilized procedure in this study, which is explained in the following sections.

**Table 1:** Characteristics of datasets involved in the analysis and validation

Dataset	Platform	Number of Samples
Datasets for main analyses		
GSE29312	Illumina HumanHT-12 V3.0 expression beadchip (GPL6947)	Normal: 9 ACs: 20 HAM/TSP: 10
GSE29332	Illumina HumanWG-6 v3.0 expression beadchip (GPL6884)	Normal: 8 ACs: 17 HAM/TSP: 10
GSE55851	Agilent-026652 Whole Human Genome Microarray 4 × 44 K v2 (GPL10332)	Normal: 3 ACs: 6 ATLL: 12
GSE33615	Agilent-014850 Whole Human Genome Microarray 4 × 44 K G4112F (GPL4133)	Normal: 21 ATLL: 52
Datasets for validation		
GSE38537	Agilent-014850 Whole Human Genome Microarray 4 × 44 K G4112F (GPL6480)	ACs: 4 HAM/TSP: 4
GSE43017	Affymetrix Human Genome U133 Plus 2.0 Array (GPL570)	ATLL: 7

### Determination of differential co-expressed modules

To find the differential co-expressed modules among two conditions, the DiffCoEx algorithm was employed [13]. DiffCoEx principally uses Weighted Gene Co-expression Analysis (WGCNA) [12] and contains five steps as follows: (1) An adjacency matrix is constructed for each condition in which their elements are the calculated correlations between each gene pairs. We used Pearson coefficient to measure correlations. (2) A matrix of adjacency difference (d_ij_) is determined by calculating the signed squared correlation coefficients. The higher d_ij_ values are considered as significant co-expression changes between two genes. The soft threshold parameter β is determined so that the network follows a scale-free topology [15]. Then, d_ij_ values reach the power of β. (3) The topological overlap dissimilarity matrix (dissTOM) is constructed to detect the shared genes with common neighbors in the graph. It is constituted based on the adjacency matrix and contains the differential correlation network. (4) The hierarchical clustering is performed with flashClust [16]. Then, dynamicTreeCut function is used to extract gene groups (modules) from the obtained dendrogram and mergeCloseModules function is employed to merge close modules. Each module is specified with a color. (5) The statistical significance of differential co-expression is evaluated using the dispersion statistic to quantify the correlation alteration between two conditions. In this study, we identified differential co-expressed modules between normal and ACs, ACs and ATLL, ACs and HAM/TSP. Moreover, module-to-module co-expression changes were examined by evaluating the significance of the correlation alterations among the genes in each module pair. For this purpose, a similar “module-to-module” dispersion measure was determined and null distributions were produced from the same permutation approach.

### Pathway enrichment analysis

The most connected genes in each module were enriched in the KEGG utilizing g:Profiler webtools (version: 1185_e69_eg16) [17] and Enrichr. The common gene expressions between ACs, ATLL, and HAM/TSP groups were considered as the background. The relevant terms with Benjamini–Hochberg FDR < 0.05 were employed as statistically significant.

## Results

### Identification of differential co-expressed modules

**Fig. 2:**
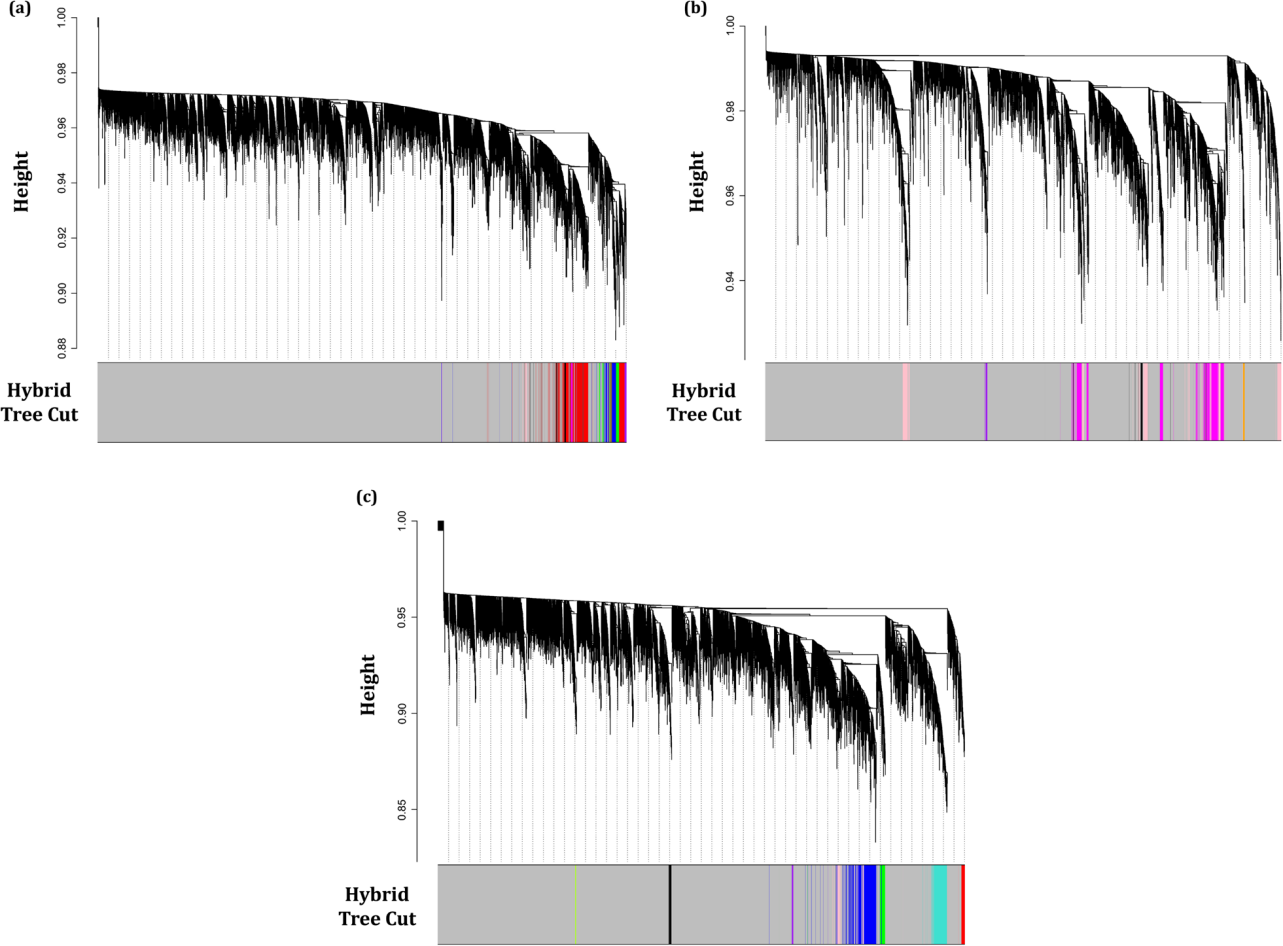
Dendrogram of genes clustered based on (1-TOM) with assigned module colors of **a** ACs, **b** ATLL, and **c** HAM/TSP. The colored rows show the module membership acquired after merging modules by the dynamic tree cut method

**Fig. 3:**
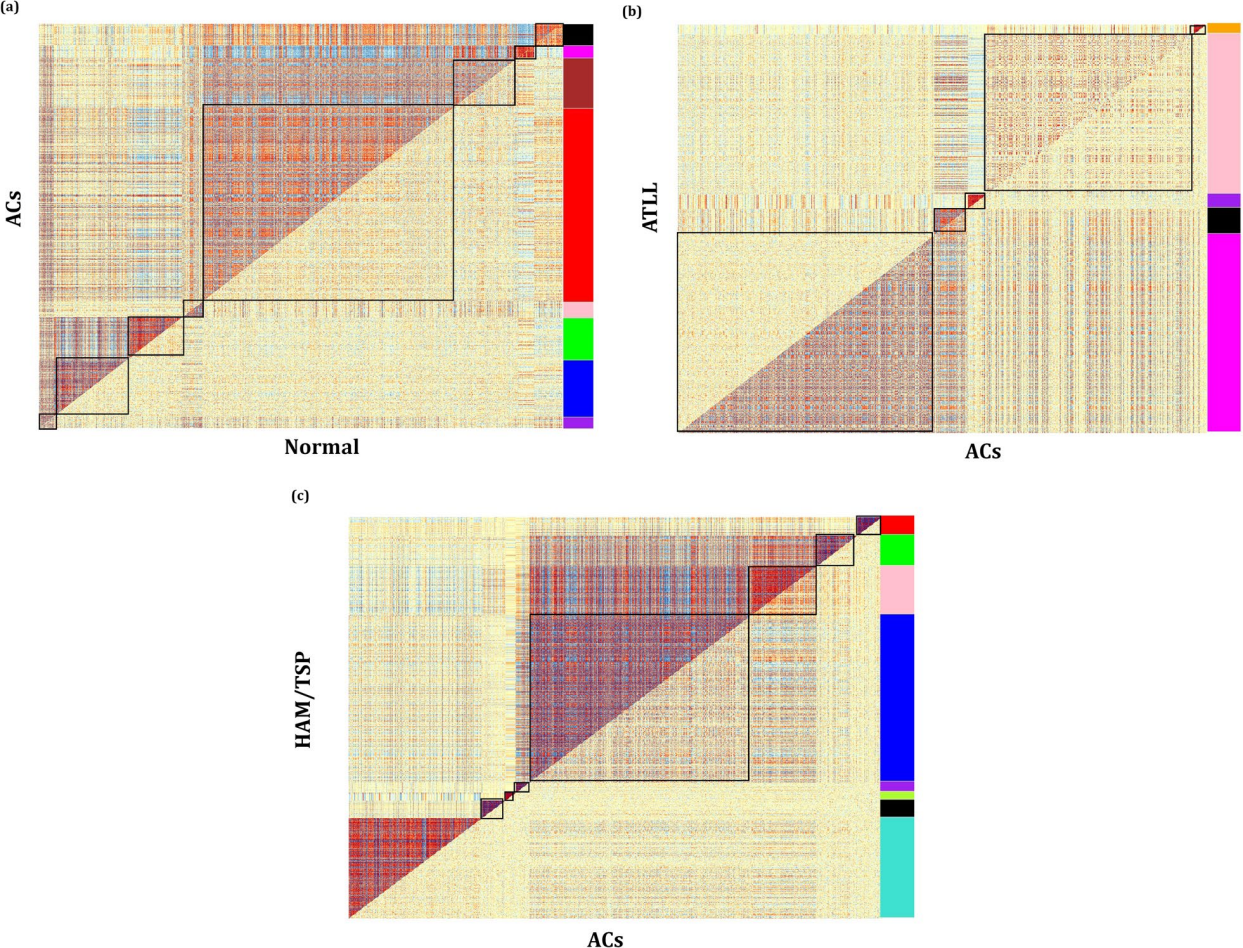
Comparative correlation heatmap containing differentially co-expressed modules between **a** normal and ACs, **b** ACs and ATLL, and **c** ACs and HAM/TSP. The upper and lower diagonals of the main matrix represent a correlation between gene pairs among each studied group. Modules are specified in the heatmap by black squares

**Fig. 4:**
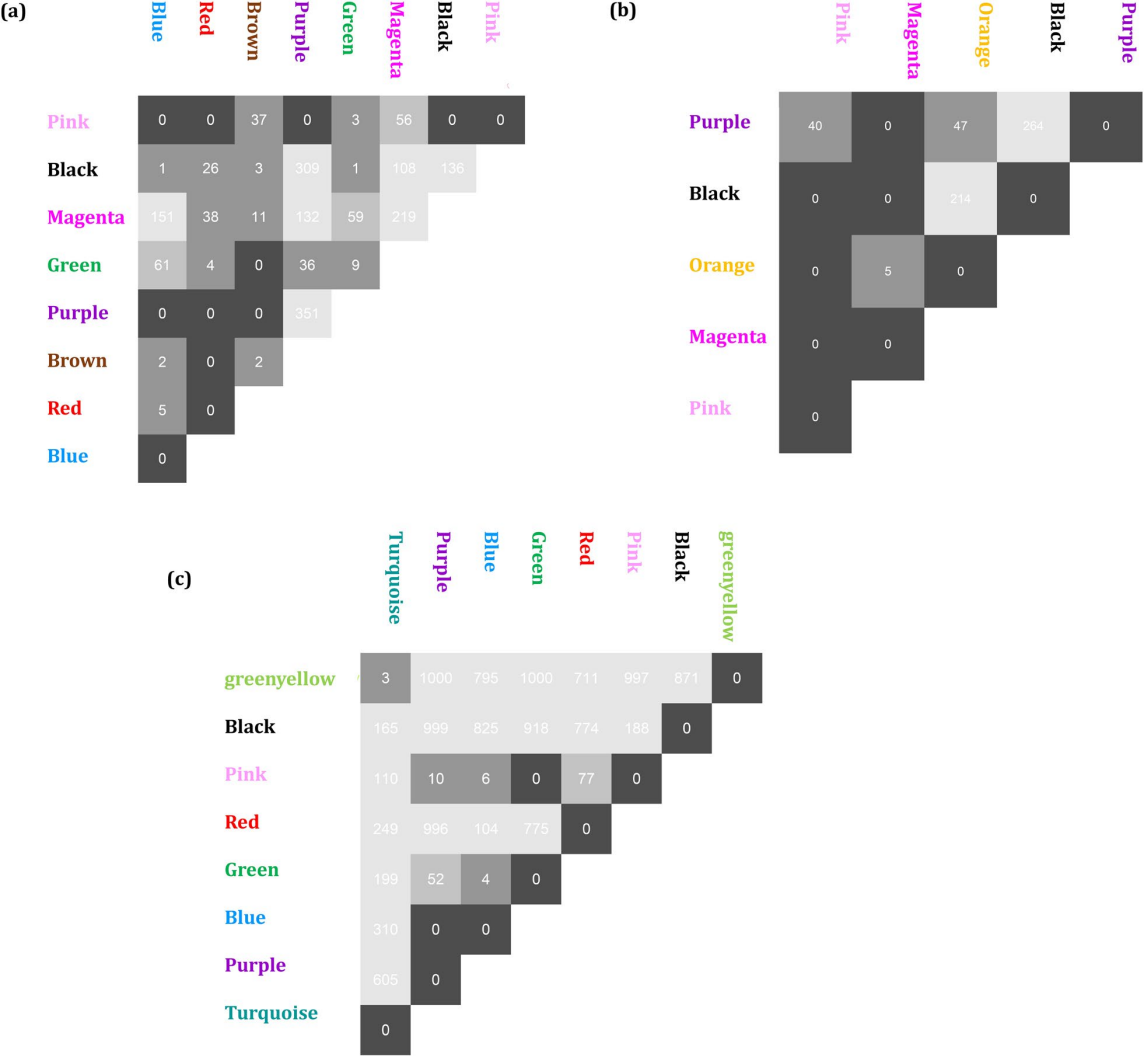
Module-to-module co-expression changes using permutations for **a** DiffCoEx_NA, **b** DiffCoEx_AA, and **c** DiffCoEx_AH. This figure is the result of the significance analyses. After 1000 permutations of the samples between every two conditions, the dispersion values were computed for each module and every possible module pair. The number in each cell shows the p-value (number/1000) identified for the within-module co-expression change. Black cells show significant p-value and light grey modules represent insignificant *p* value

The DiffCoEx algorithm was employed to find the differential co-expressed gene modules between different sample groups. Through this algorithm, the co-regulated gene clusters that differentially co-expressed between two groups were determined. After computing Pearson correlation and then adjacency differences matrices according to Methods, the topological overlap matrices (TOMs) were constructed with β power of 2, 3, and 2 for Normal-ACs, ACs-ATLL, and ACs-HAM/TSP, respectively. These values are the lowest power for which the scale-free topology fit index reaches 0.80. Afterward, the differential co-expressed modules were identified in each comparative group utilizing hierarchical clustering and then merging adjacent clusters (Fig. 2a–c). Each module is specified with a distinctive color. The differences between the correlation pattern in each module are demonstrated in Fig. 3. A total of 8 differentially co-expressed modules were identified between Normal and ACs (DiffCoEx_NA) (Fig. 3a). Among them, the modules including brown, red, green, and blue are highly correlated in ACs samples and only pink module is highly correlated in Normal samples. Three of the five identified DiffCoEx modules between ACs and ATLL groups (DiffCoEx_AA) including orange, pink, and purple modules are mostly correlated in ATLL and the magenta module pursues the opposite pattern (Fig. 3b). All eight significant differential modules identified between ACs and HAM/TSP (DiffCoEx_AH) are highly correlated in HAM/TSP (Fig. 3c). The list of the identified genes in each module is mentioned in Additional file 1. Moreover, the module to module co-expression alterations related to each comparison group was determined by employing permutations. This matrix reveals the correlations between the differentially co-expressed modules. To this end, 1000 sample permutations across the two conditions were carried out. Afterward, the correlation alteration for each gene group (dispersion value) was calculated for each module pair. Figure 4 shows the module-to-module co-expression alterations across DiffCoEx_NA, DiffCoEx_AA, and DiffCoEx_AH, in which the within-module dispersion value for each module with permuted data than with original data is specified with p-value (the numbers in each cell divided to 1000) [13]. Figure 4a discloses that although there is no obvious differential correlation in black, magneta, and purple modules between ACs and normal groups, they were determined as a differentially co-expressed module due to their significant correlation with the genes in other differentially co-expressed modules (correlation of black and magenta with brown and red modules as well as correlation of purple with blue and green modules). Likewise, the black module in DiffCoEx_AA has a remarkable correlation with the genes in magenta (Fig. 4b) as well as pink and blue modules in DiffCoEx_AH with the green module (Fig. 4c). In order to find the most connected genes at the protein levels, modules were submitted to STRING and the modules with unconnected proteins were excluded. Therefore, modules brown, red, and blue in ACs, pink and purple in ATLL, and blue, turquoise, and pink in HAM/TSP were selected (Additional file 1).

### Validation of modules in the external dataset

In order to evaluate the reliability of the identified differential co-expressed modules, their preservations in an independent dataset were surveyed. For this purpose, the function of “modulePreservation” was used to measure Z_summary_ and medianRank through a permutation test (200 times) [18]. Z_summary_ score is half the sum of the mean of Z_density_ and Z_connectivity_. MedianRank is a rank-based scale that depends on an observed preservation statistic and is not dependent on module size [19]. A lower medianRank score reveals the high preservation of the module. The module preservation analysis was employed to validate the ACs and HAM/TSP modules in GSE38537 and ATLL modules in GSE43017. Modules with a *Z*_summary_ > 4 and medianRank < 8 were regarded as moderate-high preserved modules [20, 21]. The results are mentioned in Additional file 2. Therefore, module brown from ACs and module purple from ATLL with *Z*_summary_ < 4 were excluded.

### Pathway enrichment analysis

The further pathway enrichment analysis clarified that modules related to ACs were generally entered in pathways related to viral infection, Immune system, cancer, and inflammation like Human T-cell leukemia virus 1 infection, Toll-like receptor signaling pathway, Pathways in cancer, PI3K-Akt signaling pathway, Chemokine signaling pathway, Cell adhesion molecules (CAMs), NOD-like receptor signaling pathway, Th17 cell differentiation, Th1 and Th2 cell differentiation, JAK-STAT signaling pathway, and MAPK signaling pathway (Additional file 3, Sheet 1).

The identified differential modules in ATLL were mostly enriched in cancer-, viral- and immune-related pathways such as PI3K-Akt signaling pathway, Chemokine signaling pathway, Antigen processing and presentation, Viral carcinogenesis, Pathways in cancer, cAMP signaling pathway, Proteoglycans in cancer, Hippo signaling pathway, Th1 and Th2 cell differentiation, Th17 cell differentiation, ErbB signaling pathway, and VEGF signaling pathway (Additional file 3, Sheet 2). Moreover, the selected proteins in DiffCoEx_AH were enriched in viral infection, immune system, inflammation, and neurological pathways like Human T-cell leukemia virus 1 infection, PI3K-Akt signaling pathway, Th17 cell differentiation, Th1 and Th2 cell differentiation, MAPK signaling pathway, Apoptosis,, NF-kappa B signaling pathway, NOD-like receptor signaling pathway, Sphingolipid signaling pathway, JAK-STAT signaling pathway, Chemokine signaling pathway, Parkinson disease, and Neurotrophin signaling pathway (Additional file 3, Sheet 3). The results show that the immune and viral-related pathways are activated in ATLL and HAM/TSP, however, cancer pathways are mostly activated in ATLL and neurological pathways in HAM/TSP.

### Specific genes-pathways

**Fig. 5:**
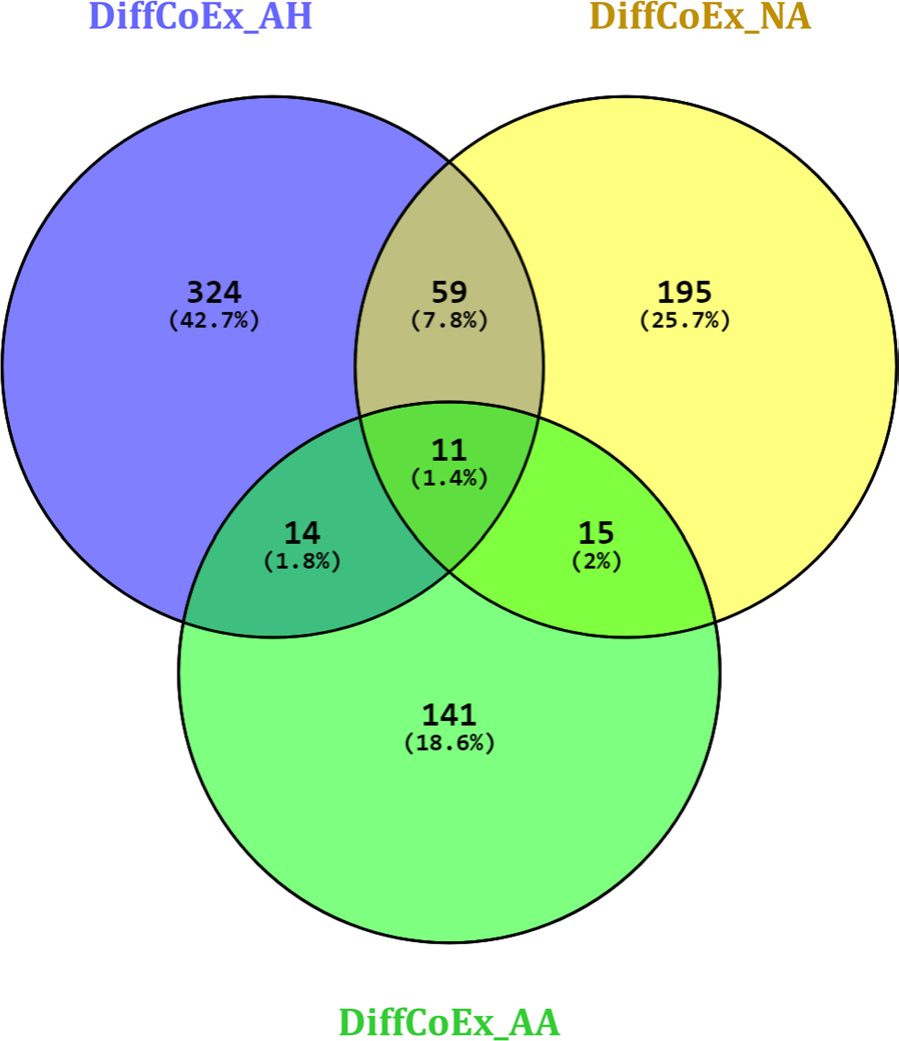
Venn diagram representing the number of common and specific pathways-genes in each DiffCoEx group

Although the identified modules were almost enriched in similar pathways, different genes were involved in the dysregulation of these pathways in each disease. To elucidate the specific genes, which may significantly dysregulate a biological pathway in each disease state, the unique pathways-genes were explored. Venn diagram depicted in Fig. 5 demonstrates the number of common and specific pathways-genes in each DiffCoEx group. The list of specific pathways-genes is also mentioned in Additional file 4. As the figure indicates, some pathways are activated after virus infection and also in two diseases. On the other hand, pathways including Proteoglycans in cancer and Rap1 signaling pathway are mostly dysregulated in ATLL as well as Sphingolipid signaling pathway, DNA replication, Parkinson disease, and Neurotrophin signaling pathway in HAM/TSP. These pathways considering their specific dysregulated genes can be furthermore studied to design proper treatment for ATLL or HAM/TSP.

### Finding potential biomarkers

In order to find potential biomarkers for each disease, the common genes between identified DiffCoEx and differentially expressed genes (DEGs) were found. To this end, DEGs between ACs and ATLL (DEGs_AA) as well as ACs and HAM/TSP (DEGs_HA) considering adj.p.val < 0.05 and |log FC|> 1 were found. Therefore, the shared genes between module pink in ATLL and DEGs_AA, as well as common genes between blue, pink, and turquoise modules in HAM/TSP and DEGs_HA, were identified. Eventually, the specific genes that were uncommon between ATLL and HAM/TSP were determined. As a result, KIRREL2, RAB36, and KANK1 were identified as specific genes and possible candidate biomarkers for HAM/TSP. Moreover, 77 specific genes were found for ATLL. To find the most important gene players in the progression of ATLL, pathway enrichment analysis was performed and the pathways enriched by at least 3 proteins were identified. Four pathways including Neuroactive ligand-receptor interaction, cAMP signaling pathway, Calcium signaling pathway, and Hippo signaling pathway were enriched by 15 genes including LTB4R2, HCN4, FZD9, GRIK5, CREB3L4, TACR2, FRMD1, LHB, FGF3, TEAD3, GRIN2D, GNRH2, PRLH, GPR156, and CRHR2. These genes may also be proposed as potential biomarkers for ATLL (Additional file 5).

## Discussion

**Fig. 6:**
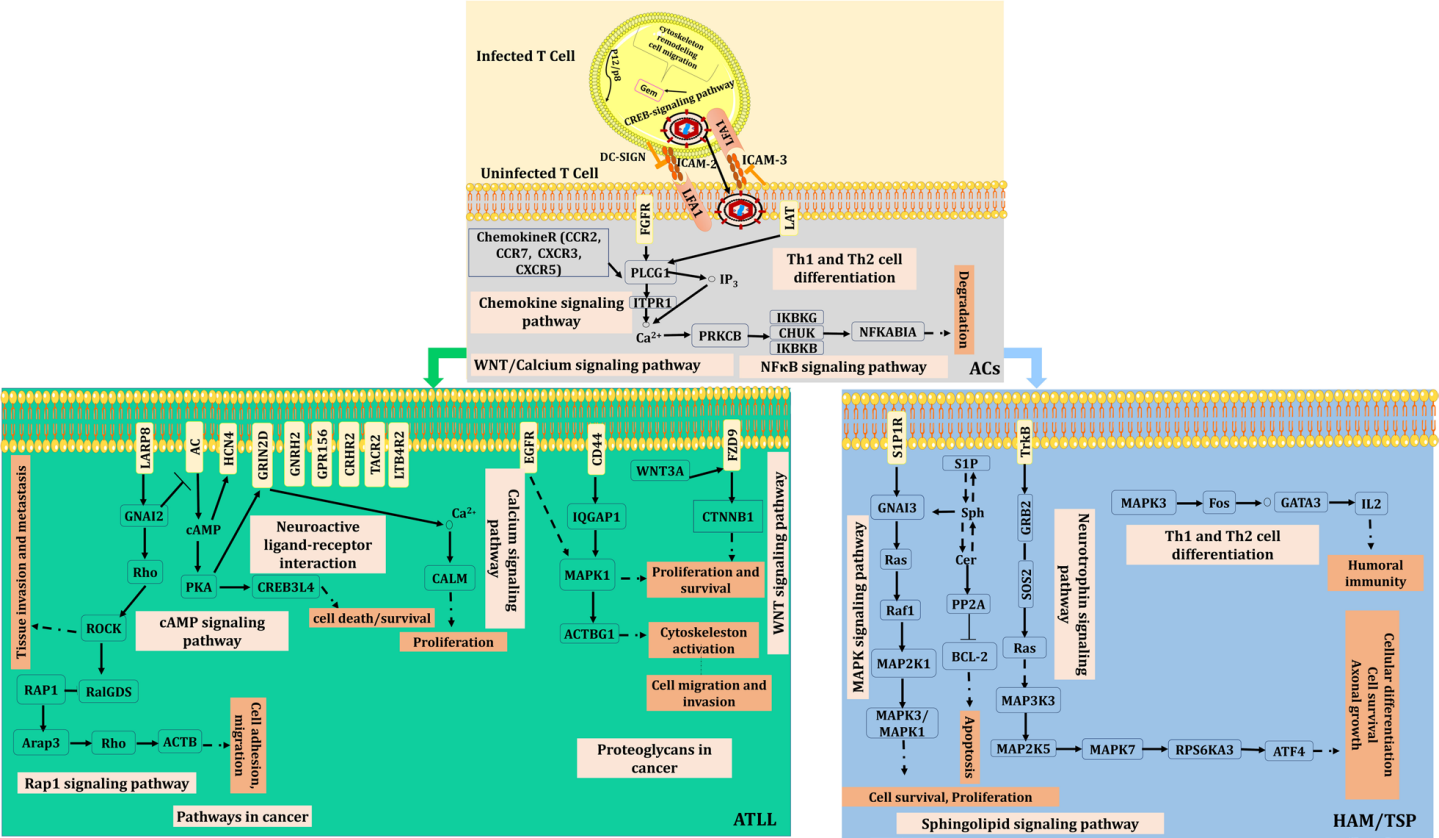
The proposed signaling networks implicated AC state and pathogenesis of ATLL and HAM/TSP diseases

The functional cellular pathways implicated in the HTLV-1 infected ACs and two main HTLV-1 related diseases (including ATLL and HAM/TSP) have not been completely figured out. Herein, we tried to shed light partially on the differences between the involved genes and also activated pathways between these diseases and AC state through a differential co-expression analysis. To this end, we applied DiffCoEx algorithm to find differential co-expressed genes between normal and ACs, ACs and ATLL, ACs and HAM/TSP. The most important activated pathways in each condition are depicted as a schematic signaling network in Fig. 6.

While the majority of the infected subjects by HTLV-1 remain as asymptomatic carriers, the most challenging question is the reason for progression from an asymptomatic viral infection toward ATLL or/and HAM/TSP. The identified differential co-regulated modules in ACs versus normal group revealed the activation of pathways related to the HTLV-1 infection which are expected.

HTLV-1 transfers between the infected and uninfected T cells through several mechanisms. One of the main mechanism is the formation of virological synapse (VS) [22]. This type of cell to cell transfer has notable advantages for viruses including more efficient transfer, elevating transfer speed, and confining the exposure to host immune defense mechanisms [23]. One of the major prerequisite steps to activate this transmission mechanism is the involvement of LFA-1 protein encoded by *ITGLA*. LFA-1 naturally binds to ICAM-1 that leads to changing both infected and uninfected cells. This provides the proper VS condition to transfer the virus. Tax and p12 implicate in the activation of LFA-1 and ICAM-1 expression [22, 24].

NF-kappa signaling is an important pathway in cell proliferation. It maintains cellular balance as well as Th1 and Th2 cell differentiation pathway through the expression alteration of the involved genes such as *IKBKG* and *PLCG1*. HTLV-1 can activate both Th1 and Th2 through interference in the NF-kappa signaling pathways based on the infection step. The downregulation of *LCK* was also observed in ACs which may be due to the expression of the viral regulatory tax gene [25]. Moreover, the obvious dysregulation of chemokine and cytokine-cytokine interaction pathways through down-regulation of CCR7 and CCR2 are also observed in this study. Our results showed that the virus in ACs keeps a balance in the successful infection by avoiding immune response. After latent infection of the virus, ATLL or HAM/TSP can be developed. Perhaps developing inflammation is the substantial difference between HAM-TSP and ACs.

HAM/TSP is known as an inflammatory disease in which several immune cells together have critical roles in the disease progression. The pathogenesis of HAM-TSP is explained in three levels: peripheral, transmit to the central nervous system, and damage to the nervous tissues. It appears that multiple genes and pathways are activated at each level which has been shown in this work.

Sphingolipid is a regulator of the cell signals through two secondary lipid messengers including ceramide (Cer) and sphingosine-1-phosphate (S1P). Cer stimulates apoptosis in the oligodendrocytes and neurons, and S1P speeds up the exit of lymphocytes from lymphoid tissues to blood [26]. Sphingolipid signaling pathway is affected in HAM/TSP that reveals the importance of theses metabolites and genes of *MAPK8, PPP2R5E, GNAI3, S1PR1, MAPK1, RAF1, BID, NSMAF, TNFRSF1A, MAPK3* in the progression of disease.

Cer activates PP2A (PPP2R5E belongs to this protein family) and BID to stimulate apoptosis [27]. It also targets the kinase suppressor Ras which aids to regulate TNFα-mediated (may by the function of TNFRSF1A) actuation of MAPK3 and MAPK1 [28, 29], activation of RAF-1, and MAPK pathway [30]. Moreover, the overexpression of S1PR1 and interaction with GNAI3 helps to activation of MAPK pathway and promotes migration and cell survival [31]. Also, NSMAF is necessary for TNF-mediated activation of neutral sphingomyelinase and probably has a function in regulating responses of TNF-induced cellular such as inflammation [32].

DNA replication is another pathway that should be considered in the development of HAM/TSP. DNA replication is increased in the Tax-expressing cells causing progression of cell cycle, even in the presence of DNA damaging agents. Therefore, DNA damage can result in the accumulation of mutations in the Tax-expressing cell and also apoptosis [33]. In our previous studies, we showed the importance of apoptosis in the HAM/TSP pathogenesis [34, 35] which also confirms in this study.

Neurotrophin signaling pathway is mostly activated in HAM/TSP. This pathway is associated with the level of neurotrophin and downstream signaling cascades. The inhibitor factors in neurotrophin signaling pathway are strongly regulated by degradation and dephosphorylation. Neurotrophin signaling pathway implicates in several neurodegenerative disorders, such as Huntington's disease, Alzheimer's disease, and psychiatric disorders like depression and substance abuse [36, 37]. In this study, the function of co-regulated of genes including RPS6KA3, MAP3K3, PSEN1, SOS2, MAP2K5, RELA, ATF4 in the activation of Neurotrophin signaling pathway in HAM/TSP were clarified. TrkA is one of the main receptors that interact with neurotrophins. It leads to the activation of intracellular signaling cascades, actuation of Ras via GRB2 and SOS2, and further promotion of the ERK/MAPK pathway with the functional activity of MAP3K3 and MAP2K5. Moreover, RPS6KA3 phosphorylates ATF4 and has critical roles in cell survival, cellular differentiation, and axonal growth [38].

Three genes comprising KIRREL2, RAB36, and KANK1 were found as biomarkers for HAM/TSP. *KIRREL2* (*NEPH3*) belongs to the immunoglobulin superfamily of cell adhesion molecules. *NEPH3* is regulated by the cooperation of WT1 and NF-κB in podocytes. *NEPH3* is a downstream target of Ptf1a in the progressing central nervous system. It is also expressed in early postmitotic neurons of the developing spinal cord. *NEPH3* intercedes the adhesion between the Ptf1a-progenitors, which is essential for maturation, neuronal migration, and differentiation [39]. The dysregulation of NEPH3 may have a role in the progression of HAM/TSP and should be furthermore studied.

*RAB36* is a member of the RAB family which is located on chromosome 22q11.2. It was reported that the depletion of RAB36 leads to G1 cell cycle arrest, disruption of mesenchymal-epithelial transition, and simplification of the malignant rhabdoid tumors dissemination [40, 41]. The information about the role of RAB36 in the development of diseases is limited, so further investigation is indispensable.

KANK1 belongs to the Kank family which is mainly involved in the cytoskeleton formation by regulating actin polymerization. The mutated KANK1 may develop the central nervous system disorder (cerebral palsy spastic quadriplegic type 2). It also inhibits the cell migration and formation of actin fiber. It can inhibit malignant peripheral nerve sheath tumors through the regulationof CXC5 as an apoptosis-related gene [42, 43].

ATLL is another disease caused by HTLV-1 infection. The overall identified DiffCoEx_AA and the activated pathways reveal that the viral infection provides a primary condition in the infected cells to malignancy in association with genetic susceptibility and other risk factors [44]. The differential co-expressed genes between ATLL and ACs implicates in cancer- and malignancy-related pathways. The final goals of these pathways are sustained angiogenesis, evading apoptosis, tissue invasion, block differentiation, proliferation, and genomic instability. Form this study, the dysregulation of pathways including Rap1 signaling pathway and Proteoglycans in cancer were identified in ATLL. Rap1 signaling pathway is activated with the involvement of several proteins. Rap1 upregulates ARAP3 which is a PI3K effector. ARAP3 selectively uses Rho as its substrate, and then Rho targets ACTB resulting in cell adhesion and migration. On the other hand, the expression of WNT3A has been reported along with the WNT signaling pathway components FZD9 and CTNNB1 [45]. Moreover, IQGAP1 is a significant scaffold in the EGF-stimulated MAPK cascade due to its direct interaction with MAPK1 and EGFR [46]. These events ultimately result in proliferation, cell migration and invasion, and also cytoskeleton activation as parts of the pathway of Proteoglycans in cancer.

Fifteen genes including LTB4R2, HCN4, FZD9, GRIK5, CREB3L4, TACR2, FRMD1, LHB, FGF3, TEAD3, GRIN2D, GNRH2, PRLH, GPR156, and CRHR2 were also determined as potential biomarkers for ATLL.

The importance of cAMP in developing hematological cancer has been reported previously [47]. The cAMP levels are regulated by the balance between the functions of two enzymes: adenylyl cyclase (AC) and cyclic nucleotide phosphodiesterase (PDE) [48]. cAMP binds to HCN4 and also mediates phosphorylation of proteins by PKA. PKA may increase the NMDAR currents (GRIN2D is a subunit of NMDA receptors). The overexpression of GRIN2D may cause an excessive influx of Ca^2+^ and as a result the regulation of CALM1 in the calcium signaling pathway [49]. On the other hand, regulation of transcription by PKA is obtained by direct phosphorylation of the CREB which finally may lead to cell survival/death [50].

TEAD3 is a transcription factor and FRMD1 is an activating transcription factor binding which have important functions in the Hippo signaling pathway, a pathway involved in tumor suppression by limiting proliferation and boosting apoptosis.

FGF3 belongs to the basic fibroblast growth factor (FGF) gene family which plays a major role in cell differentiation and proliferation and is involved in the calcium signaling pathway [51]. Furthermore, the contribution of GNRH2, PRLH, GPR156, GRIK5, LHB, and CRHR2 to the neuroactive ligand-receptor interaction pathway are specified in ATLL in consistent with some other cancers [52, 53].

LTB4R2 is one of the LTB4 receptors that is associated with invasion, survival, and metastasis. TACR2 is also a receptor for tachykinins that is mainly expressed in the periphery including inflammatory cells. Overexpression of TACR2 can develop the proliferation and migration of cancer cells by regulating the Wnt signaling pathway [54]. It seems that further in vivo and in vitro studies on the function and the involved proteins in this pathway may introduce a novel therapeutic way.

Our results reveal that more dysregulated genes and pathways are involved in the HAM/TSP pathogenesis in comparison to ATLL, which probably explains more prevalence of ATLL in the HTLV-1 infected patients [55]. Interestingly, some features which are implicated the HTLV-1 infection pathway and activated by differential co-expressed genes between HAM/TSP and ACs, do not observe in ATLL. Given these results, it seems that the host features and genetic susceptibility are the main players in ATLL. Moreover, the specific identified genes for each condition have of importance in the future proposed treatment for each disease.

## Conclusion

The pathogenesis mechanism and development of diseases caused by HTLV-1 infection is obscure. Herein, through the identification of differential co-expressed module analysis and pathway enrichment, the major genes implicated the pathogenesis process of ACs toward ATLL and HAM/TSP were clarified. It seems that different dysregulated genes cause the activation of similar pathways in ATLL and HAM/TSP. They may be proposed as potential therapeutic targets. However, further studies should be designed and performed to evaluate the identified genes in each condition.

## Supplementary Information

**Additional file 1**. List of the identified differential co-expressed modules in ACs, ATLL, and HAM/TSP.

**Additional file 2**. The preservation of modules in the external datasets (ACs, ATLL, and HAM/TSP).

**Additional file 3**. The enriched pathways by the connected proteins in each identified DiffCoEx_NA, DiffCoEx_AA, and DiffCoEx_AH.

**Additional file 4**. The list of specific pathways-genes in ACs, ATLL, and HAM/TSP.

**Additional file 5**. The list of DEGs, common between DEGs and DiffCoEx_AA, and specific genes for ATLL.

## Data Availability

All data generated or analyzed during this study are included in this published article [and its supplementary information files].

## Abbreviations

HTLV-1: Human T-cell Leukemia Virus type-1
ATLL: Adult T-cell Leukemia/Lymphoma
HAM/TSP: HTLV-1 Associated Myelopathy/Tropical Spastic Paraparesis
ACs: Asymptomatic carriers
DiffCoEx: Differentially co-expressed modules
TOMs: Topological overlap matrices
DEGs: Differentially expressed genes
